# Correlations between Risk Factors for Breast Cancer and Genetic Instability in Cancer Patients—A Clinical Perspective Study

**DOI:** 10.3389/fgene.2017.00236

**Published:** 2018-02-16

**Authors:** Márcia Fernanda Correia Jardim Paz, Marcus Vinícius Oliveira Barros de Alencar, Antonio Luiz Gomes Junior, Keylla da Conceição Machado, Muhammad Torequl Islam, Eunus S. Ali, Manik Chandra Shill, Md. Iqbal Ahmed, Shaikh Jamal Uddin, Ana Maria Oliveira Ferreira da Mata, Ricardo Melo de Carvalho, Kátia da Conceição Machado, André Luiz Pinho Sobral, Felipe Cavalcanti Carneiro da Silva, João Marcelo de Castro e Souza, Daniel Dias Rufino Arcanjo, Paulo Michel Pinheiro Ferreira, Siddhartha Kumar Mishra, Juliana da Silva, Ana Amélia de Carvalho Melo-Cavalcante

**Affiliations:** ^1^Postgraduate Program in Pharmaceutical Sciences, Federal University of Piauí, Teresina, Brazil; ^2^Biomedicine Department, UNINOVAFAPI University, Teresina, Brazil; ^3^Department of Pharmacy, Southern University Bangladesh, Chittagong, Bangladesh; ^4^School of Medicine, Flinders University, Adelaide, SA, Australia; ^5^Department of Pharmaceutical Sciences, North South University, Dhaka, Bangladesh; ^6^Pharmacy Discipline, Life Science School, Khulna University, Khulna, Bangladesh; ^7^University Hospital, Federal University of Piauí, Teresina, Brazil; ^8^Department of Biological Sciences, Federal University of Piauí, Picos, Brazil; ^9^Department of Biophysics and Physiology, Universidade Federal do Piauí, Teresina, Brazil; ^10^Cancer Biology Laboratory, School of Biological Sciences (Zoology), Dr. Harisingh Gour Central University, Sagar, India; ^11^Program in Cellular and Molecular Biology Applied to Health Sciences, Universidade Luterana do Brasil, Canoas, Brazil

**Keywords:** breast cancer, risk factors, genetic instability, apoptosis, chemotherapy, toxicogenomics

## Abstract

Molecular epidemiological studies have identified several risk factors linking to the genes and external factors in the pathogenesis of breast cancer. In this sense, genetic instability caused by DNA damage and DNA repair inefficiencies are important molecular events for the diagnosis and prognosis of therapies. Therefore, the objective of this study was to analyze correlation between sociocultural, occupational, and lifestyle risk factors with levels of genetic instability in non-neoplastic cells of breast cancer patients. Total 150 individuals were included in the study that included 50 breast cancer patients submitted to chemotherapy (QT), 50 breast cancer patients submitted to radiotherapy (RT), and 50 healthy women without any cancer. Cytogenetic biomarkers for apoptosis and DNA damage were evaluated in samples of buccal epithelial and peripheral blood cells through micronuclei and comet assay tests. Elder age patients (61–80 years) had higher levels of apoptosis (catriolysis by karyolysis) and DNA damage at the diagnosis (baseline damage) with increased cell damage during QT and especially during RT. We also reported the increased frequencies of cytogenetic biomarkers in patients who were exposed to ionizing radiation as well as for alcoholism and smoking. QT and RT induced high levels of fragmentation (karyorrhexis) and nuclear dissolution (karyolysis) and DNA damage. Correlations were observed between age and karyorrhexis at diagnosis; smoking and karyolysis during RT; and radiation and karyolysis during QT. These correlations indicate that risk factors may also influence the genetic instability in non-neoplastic cells caused to the patients during cancer therapies.

## Introduction

The International Agency for Research on Cancer (IARC) and the World Health Organization (WHO) published in the World Health Report 2014 with the prediction of 27 million new cancer cases by the year 2030 resulting in 17 million deaths and 75 million people living with cancer annually. Cancer is one of the major public health problems worldwide (Vaidya et al., 2015; Buyukavcu et al., 2016), occupying the second position of global incidence (Buyukavcu et al., 2016). In Brazil, approximately 600,000 new cancer cases every year are estimated (INCA, 2016).

Breast cancer is a multifactorial disease with more incidences in elderly patients (Arraras et al., 2016). According to literature, more than 1.7 million cases are diagnosed worldwide (Abdel-Zaher and Eldeib, 2016), where one in eight women will probably develop breast cancer (Sismondi et al., 2015). Recent data has pointed out the association between genetic instability and cancer on the prognosis and disease progression (Collins et al., 2014). The mechanisms associated are telomere damage, centrosomal amplification, epigenetic modifications, and genotoxic DNA damage. These genomic alterations may lead to aneuploidy, induction of mutations, apoptosis, and necrosis (Bonassi et al., 2011; Burrel and Swanton, 2014; Ferguson et al., 2015). Several factors increased the risk of genetic instability in cancer development such as age, endocrine/reproductive history, behavioral/environmental factors, smoking, alcoholism, exposure to ionizing radiation, and hereditary factors (Adami et al., 2008; Mary et al., 2011; Buyukavcu et al., 2016).

Many cancer cells exhibited aneuploidies and chromosomal alterations leading to genetic instability which is a hallmark of cancer. Genetic heterogeneity is a problem for cancer therapies especially therapies targeting specific molecules. Thus, understanding the pathophysiology of genetic makeup is required to advance the prevention and cure of cancer (Tanaka and Hirota, 2016). Biomonitoring of molecular alterations can be an important tool for better understanding of cancer biology which not only secure more accurate diagnoses but also ensure treatment success of cancer (Abramczyk and Brozek-Pluska, 2016). For this, the comet assay is increasingly being used for the detection of genotoxicity (Enciso et al., 2015) as well as the micronucleus test to evaluate chromosomal mutagenesis in eukaryotes such as clastogenesis (loss of chromosome fragments) and aneugenesis (loss or gain of whole chromosomes) and cell death (Fenech and Bonassi, 2011).

Clinical and epidemiological studies are current priorities for understanding breast cancer heterogeneity especially aspects related to tumor etiology, chemoprophylaxis, and therapies, which are important strategies to improve prevention, diagnosis, prognosis and therapy efficacy (Barnard et al., 2015; Abdel-Zaher and Eldeib, 2016). Thus, because of the influence of several risk factors for the etiology of breast cancer as a confounding element in genetic instability analysis induced during cancer therapies. The present study aimed to evaluate the correlations between sociocultural, occupational and life style risk factors with the levels of genetic instabilities during the first diagnosis and during chemotherapy (QT) and radiotherapy (RT). This study used toxicological biomarkers as indicative of nuclear abnormalities and genotoxicity in non-neoplastic cellular samples of the buccal epithelium and peripheral blood of breast cancer patients.

## Materials and Methods

### Individuals

All patients were informed of the procedures and provided a written informed consent prior to sample collection. This study was performed in compliance with the Helsinki Declaration and was approved by the ethics committee of the Centro Universitário UNINOVAFAPI, Brazil (approval number: 0406.0.043.000-11). Between 2012 and 2015, 150 individuals were attended at the São Marcos Hospital where 100 patients were diagnosed with breast cancer including 83 with invasive ductal carcinoma (83%), three with invasive lobular carcinoma (3%), three with ductal carcinoma *in situ* (3%), three with medullary carcinoma (3%), and three with phyllodes tumor (3%). A total of 50 healthy women were enrolled as controls. All breast cancer patients were in between stage I and III based on TNM staging system (TNM classification of malignant tumors). The QT group of patients received two different QT protocols: standard FAC QT (fluorouracil 500 mg/m^2^, doxorubicin 50 mg/m^2^, cyclophosphamide 500 mg/m^2^—all i.v.) or AC-T QT (doxorubicin 50 mg/m^2^ and cyclophosphamide 500 mg/m^2^ i.v.) for 21 days including 80 mg/m^2^ of docetaxel (Taxotere) weekly for 12 weeks. Patients of RT group received 25 sessions of RT isolated or after QT with a total dose of 4500–5000 cGy (180–200 cGy/fraction). Breast cancer patients who presented renal and liver dysfunctions were excluded from this study.

### Sample Collection

Peripheral blood and buccal mucosa cells were samples and processed on the same day. Four collections were performed in breast cancer patients: (1) at the time of breast cancer diagnosis, prior to treatment; (2) at the second cycle of QT; (3) at the third week of QT; (4) at the fourth week of QT. Additionally, four collections were performed during RT: (1) before the RT; (2) at the second cycle of RT; (3) at the third week of QT; (4) 21 days after the last RT session. Blood collection was performed on the non-mastectomized side arm vein. For the Micronucleus Test, samples of oral mucosa were obtained by means of cytobrush brushes and stored in microtubes with 0.9% saline, duly identified. The samples were kept under refrigeration (4°C) until processing of the Micronucleus Test and Comet Assay at the Laboratory of Toxicological Genetics of the Federal University of Piauí—UFPI which served as support for the experiments.

### Survey

The questionnaire for the *International Commission for Protection against Environmental Mutagens and Carcinogens* (ICPEMC) proposed by Carrano and Natarajan (1988) was applied during the monitoring of cancer patients during diagnosis and cancer therapies (QT and RT). The questionnaire was related to various risk factors such as age, lifestyle, environmental, and occupational exposure aspects, family history, diseases, and nutritional factors. It was adapted to meet the needs of the study as well as to include information provided by patients during anamnesis including the use of alternative therapies as a therapeutic source during cancer treatment. The various risk factors were observed according to their frequencies and statistically correlated with genetic instability biomarkers such as DNA damage and apoptosis in non-neoplastic cells of patients before and during QT and RT.

### Genomic Instability Analysis

#### Comet Assay in Peripheral Blood Cells

After blood collection, samples were immediately processed according to Singh et al. (1988) with modifications. The results were expressed as damage index (DI) and frequency of damage (DF). The DI was obtained by evaluating the tail type which was classified from 0 to 4 (50 cells per slide in duplicate, i.e., 100 per individual). Class 0 (C0, genetic material without damage or intact); class 1 (C1, mild damage); class 2 (C2, moderate damage); class 3 (C3, severe damage); and class 4 (C4, maximum damage). In order to determine DI, the total score for each individual was between 0 and 400 arbitrary units, and was defined as: DI: 0 × (C0) + 1 × (C1) + 2 × (C2) + 3 × (C3) + 4 × (C4). Each image was scored according to the extent of DNA migration based on a visual analysis in 100 cells of each slide. The DF analysis, varying from 0 to 100 (%), was define as: DF = 100 - C0, in which C0 represents the number of class 0 cells out of 100 cells evaluated.

#### Micronucleus Test in Buccal Epithelial Cells

The micronucleus test in buccal epithelial cells was performed according to Thomas et al. (2009). The incidence of micronuclei and nuclear abnormalities representing cell death (karyorrhexis and karyolysis) were observed in 2000 cells per patient using an optical microscope (Olympus, CX, United States) at 1000× magnification.

### Statistical Analysis

Statistical analysis was performed using SPSS version 13.0 (IBM Corp., Armonk, United States) for the analysis of variance with the non-parametric one-way ANOVA test and Spearman correlations. Kruskal–Wallis test was used to compare the hematological and biochemical parameters and the Student’s *t*-test for comparisons between the exposed and non-exposed individuals in the genotoxic evaluation. Significance levels of up to *p* < 0.05 were adopted.

## Results and Discussion

### Patients Characteristics

Socioeconomic factors, environmental and behavioral risks, psychosocial, educational and occupational aspects, lifestyle and stress levels have influenced the risk of breast cancer (Mary et al., 2011; Peretti-Watel et al., 2016). Ethnicity is an important factors for the etiology of breast cancer (Chen et al., 2002). As observed in the study, the vast majority (70–80%) of cancer patients reported were of Caucasian ethnicity (**Table 1**). Studies indicate that the incidence of breast cancer is 124 cases per 100,000 white women and 113 cases per 100,000 black women (American Cancer Society [ACS], 2009).

**Table 1:** Sociocultural characterization of occupational risks, lifestyle for breast cancer in non-diagnosed patients.

Features	Control group (*n* = 50)	Chemotherapy (*n* = 50)	Radiotherapy (*n* = 50)
**Sociocultural**			
*Etnia*			
Caucasian	50%	78%^∗^	82%^∗^
Black	34%	22%	16%
Other	16%	–	2%
*Age*^a^	48.3 ± 13.4	49.8 ± 10.6	50.4 ± 11.9
**Occupational risks**			
*Working time*			
1–2 years	8%	6%	6%
2–4 years	14%	6%	–
4–8 years	6%	4%	6%
More than 8 years	72%	84%	88%
**Working place**			
Home	64%	62%	48%
School	24%	14%	16%
Farm	12%	24%	36%^∗^
**Chemical exposure**			
Agrotoxics	–	28%^∗^	24%^∗^
Formalin	–	10%	18%
Others	–	38%	24%
None	100%	24%	30%
**Detergent exposure**			
Yes	–	92%	86%
No	–	8%	14%
**Hypochlorite exposure**			
Yes	98%	86%	80%
No	2%	14%	20%^∗^
**Life style**			
*Smoke*			
Yes	–	46%	48%
No	100%	54%	52%
**Alcohol**			
Yes	–	28%	24%
No	100%	72%	76%
**X-ray exposure**			
Yes	32%	90%^∗^	100%^∗^
No	68%	10%	


*^a^Values are the mean ± standard deviation. ^∗^*p* < 0.05 compared to the control group.*

Reproductive age involving events like menarche, menopause and pregnancy, hormone therapy confers risks that can trigger neoplasms (Patterson et al., 2013) and estrogen alterations (Monninkhof et al., 2009).

As observed in the present study, the mean age of the patients was 50 years, with no statistical differences between the groups. An earlier case study on Korean women (*n* = 1026) indicated that the mean age of breast cancer diagnosis was 46.8 years with 14.4 and 26.3 as the age of menarche and first parturition (Kang et al., 2014). However, other studies indicated that 2–20% of women diagnosed with breast cancer were under the age of 35 years with tumors that present aggressive biological behavior resulting in mortality and metastasis (Kallel et al., 2015). Moreover, women’s reproductive life, precocious menarche, nulliparity, age of first gestation over 30 years, oral contraceptives, late menopause, and hormone replacement therapy are also well established in relation to breast cancer development (Thuler, 2003; Pinho and Coutinho, 2007; Brasil, 2008, 2009). Although it is not well documented whether race and cultural differences impart differently on breast cancer induction and progression, yet these factors may have individual roles.

Breast cancer patients are also reported to have exposure to various genotoxic and carcinogenic agents in their domestic activities (detergents and hypochlorites) and occupational contaminants (pesticide) as well as exposure to ionizing radiation before and after diagnosis. Ionizing radiation leads to mutations due to increased chromosomal aberrations and alterations in DNA repair (Mozdarani, 2012) as well as RT-associated alterations including circulatory problems and secondary cancer (Hamada and Fujimichi, 2014). Approximately 50% of RT patients also reported with a smoking habit which is an identified carcinogenic agent with evident increased risk of breast cancer in humans (International Agency for Research on Cancer [IARC], 2015). Similar data (30%) were observed for the consumption of alcoholic beverages, even moderately, may be associated with the risk of breast cancer (Thuler, 2003).

### Association between Age and Genetic Instability as an Indicative of Apoptosis and Genotoxicity

Before the cancer treatment as well as during RT, nuclear abnormalities (cell death) were statistically increased in patients between 20 and 40 years of age when compared to others. Similar results were found in the range of 41–60 years in relation to the range of 61–80 years age groups indicating that the younger patients are more likely for apoptosis during RT (**Table 2**). Positive correlations were observed at the basal level between karyorrhexis and age in non-neoplastic cells of the buccal epithelium. Exposure to RT is also not selective with the risks of reaching non-neoplastic tissues (Chan et al., 2005; Taylor and Kirby, 2015). Although the benefits of cancer therapy are inquestionable, the safety aspects cannot be ignored, since these drugs’ mechanisms of action can have harmful effects on different tissues (Adão et al., 2012).

**Table 2:** Correlation of age with cell death (buccal epithelium) and genotoxicity (lymphocytes).

Cytogenetic damage	Control	Basal	Chemotherapy	Radiotherapy
**Buccal epithelium**				
*Karyorrhexis*		*R* = -0.479^∗∗^ (*p* = 0.010)		
20–40 years	183.9 ± 77.6	250.0 ± 97.0^ae^	319.1 ± 90.9^af^	516.1 ± 152.0^abef^
41–60 years	165.0 ± 103.3	276.6 ± 120.0^acde^	397.5 ± 121.9^acf^	509.7 ± 128.3^abef^
61–80 years	184.1 ± 104.4	222.0 ± 32.0^ac^	386.1 ± 189.3^acf^	419.2 ± 153.1^abf^
*Karyolysis*				
20–40 years	56.5 ± 27.2	87.6 ± 48.2^a^	93.4 ± 63.3^a^	381.9 ± 147.5^abdef^
41–60 years	50.6 ± 35.5	94.4 ± 47.9^a^	81.3 ± 37.7^a^	162.2 ± 67.3^abef^
61–80 years	73.0 ± 43.2	56.0 ± 21.0^a^	68.6 ± 22.7^a^	220.0 ± 151.8^abcdf^
**Lymphocytes**				
*Damage index*				
20–40 years	19.9 ± 8.2	199.2 ± 41.8^a^	181.1 ± 60.7^a^	218.1 ± 21.6^abf^
41–60 years	34.7 ± 16.4	178.2 ± 50.4^a^	185.7 ± 47.4^a^	204.5 ± 42.9^abf^
61–80 years	21.1 ± 10.6	193.4 ± 39.7^a^	179.7 ± 49.5^a^	215.9 ± 38.2^abf^
*Frequency of damage*				
20–40 years	14.4 ± 4.8	79.4 ± 19.1	90.0 ± 7.0	94.2 ± 7.8^ab^
41–60 years	16.8 ± 5.9	79.5 ± 18.5	90.0 ± 10.2	88.4 ± 6.9^ab^
61–80 years	14.8 ± 6.4	70.0 ± 13.5	87.2 ± 8.2	96.2 ± 2.5^ab^


*Values represent the mean ± standard deviation. Significance up to *p* < 0.05. ^a^Compared to the control group. ^b^Compared to the chemotherapy group. ^c^Compared to the age of 20–41 years. ^d^Compared to the age of 41–60 years. ^e^Compared to the age of 61–80 years. ^f^Compared to baseline level. ^∗∗^*p* values as compared to control group.*

During RT and QT, significant increases of karyorrhexes were observed in the buccal epithelium of older patients. Patients with RT showed significant increase in cell death in buccal epithelium as compared to patients undergoing QT. The age in association with hormone receptors status, family history, and genetic aspects may have implications on cancer therapies (Peto and Mack, 2000; Pinho and Coutinho, 2007; Lizarraga et al., 2013). Previous studies have shown that young women with triple negative breast tumors have worst prognosis regarding recurrence and mortality (Kallel et al., 2015; Reviriego et al., 2016). Endocrine factors/reproductive history is mainly related to estrogen stimulation whether endogenous or exogenous, pregnancy after 30 years, nulliparity, use of oral contraceptives (estrogen–progesterone), and postmenopausal hormone replacement therapy (estrogen–progesterone) are all associated with breast cancer incidence (International Agency for Research on Cancer [IARC], 2015; World Health Organization [WHO], 2015).

During cancer development, several pathways may cause genetic instability leading to cell proliferation, inflammation, immune response alterations, and resistance to apoptosis. Genetic instability may induce replicative immortality, cell cycle abnormalities, aneuploidy, and tetraploidy (Ferguson et al., 2015; Asatryan and Komarova, 2016). The genotoxic damage observed in lymphocytes from RT patients was also significant (*p* < 0.05) as compared to those observed on lymphocytes from patients during QT as well as from baseline and control group (**Table 2**). No significant statistical differences were observed between the age ranges in relation to genotoxic damages observed. Increased levels of DNA damage and inefficient repair mechanisms are molecular events of many pathogens including cancer (Gunasekarana et al., 2015). Detection of DNA damage is an initial step toward understanding cellular responses to genotoxic events. Then it is important to know the relationship between drug genotoxicity and checkpoint adaptation during DNA damage mitosis (Swift and Golsteyn, 2014).

### Correlations between Occupational Risks and Genetic Instabilities As Indicative of Apoptosis and Genotoxicity

Nuclear abnormalities as indicative of cell death in the oral epithelium, especially for karyorrhexis, showed increased levels in patients from QT and RT groups as compared to control. The karyorrhexes observed for domestic occupation were significant in relation to the risks of school and agricultural activities especially for patients in RT. Cell death observed in RT group was also significant in relation to QT group (**Table 3**). Previous studies have reported that occupational exposure to pesticides has effects on the frequency of micronuclei in oral mucosal cells with cytogenetic damage in somatic cells and correlations between some genotoxicity biomarkers (Bolognesi, 2003; Celik and Akbas, 2005). According to Rudel (2007), occupational exposure to chemicals in women involved in agriculture and/or industry should be monitored in epidemiological studies.

**Table 3:** Correlation of occupational risks with cell death (buccal epithelium) and genotoxicity (lymphocytes).

Cytogenetic damage	Control	Basal	Chemotherapy	Radiotherapy
**Buccal epithelium**				
*Karyorrhexis*				
Home	184.8 ± 66.3^d^	248.1 ± 69.8^acd^	368.4 ± 64.2^a^	391.5 ± 68.5^ab^
School	187.7 ± 53.4^d^	224.8 ± 37.8^a^	378.5 ± 72.2^a^	468.6 ± 65.9^ab^
Agricultural activity	147.2 ± 75.2	340.3 ± 74.4^a^	379.9 ± 77.8^a^	428.4 ± 75.7^abe^
*Karyolysis*				
Home	55.3 ± 39.8	94.7 ± 68.3^a^	98.1 ± 58.2^ac^	175.1 ± 69.3^abe^
School	50.0 ± 36.5	113.4 ± 34.2^ae^	56.1 ± 43.4^a^	174.5 ± 55.9^ab^
Agricultural activity	67.0 ± 37.9	85.5 ± 66.6^a^	90.4 ± 50.8^ac^	177.5 ± 89.9^abe^
**Lymphocytes**				
*Damage index*				
Home	26.2 ± 14.1	139.7 ± 58.8^ac^	193.9 ± 48.1^ac^	189.8 ± 62.9^a^
School	17.2 ± 8.7^d^	181.2 ± 73.0^ad^	231.7 ± 68.0^ad^	220.6 ± 64.2^a^
Agricultural activity	41.0 ± 21.9	138.5 ± 60.3^a^	178.4 ± 48.3^a^	215.2 ± 72.2^ab^
*Frequency of damage*				
Home	15.8 ± 10.2	72.4 ± 17.3^ac^	90.5 ± 11.0^a^	86.5 ± 12.7^a^
School	12.6 ± 5.2	91.4 ± 14.2^a^	91.5 ± 12.4^a^	93.2 ± 7.7^a^
Agricultural activity	19.6 ± 8.8	80.5 ± 17.0^a^	89.2 ± 10.4^a^	89.3 ± 17.2^a^


*Values represent the mean ± standard deviation. Significance up to *p* < 0.05. ^a^Compared to the control group. ^b^Compared to the chemotherapy group. ^c^Compared to individuals who answered “school.” ^d^Compared to individuals who answered “agriculture.” ^e^Compared to baseline damage.*

Basal genotoxic damages of lymphocytes in patients before QT and RT were statistically significant as compared to control. Likewise, patients with RT showed more genotoxic lymphocyte damage that QT patients. QT patients exposed to agricultural activities showed significant increase in genotoxic damage as compared to patients with home and school activities. However, this significance was not observed in the genotoxic damage to the lymphocytes of patients in radiotherapies (**Table 3**). Lymphocytes isolated from peripheral blood and exfoliated cells of the buccal epithelium are used to determine the effects of mutagens based on cytogenetic markers such as chromosomal aberrations, micronuclei, chromatid breaks, and comet assay (Faust et al., 2004; Hoffmann and Speit, 2005).

### Association between Smoking and Genetic Instability As Indicative of Cell Death and Genotoxicity

Nuclear abnormalities in patients with smoking habit prior to breast cancer diagnosis were significantly increased during QT and RT at baseline and during treatment (**Table 4**). Smoking can induce significant changes in DNA, as indicated by cytogenetic biomarkers, and increases the risk of cancer by elevating levels of micronuclei in buccal mucosa epithelial cells (Celik et al., 2003; Celik and Akbas, 2005). However, smoking did not significantly influence the DI and DF in lymphocytes in QT patients with reported smoking. However, RT patients showed increase in the genotoxicity parameters as compared to QT patients in relation to non-smokers before the diagnosis for breast cancer.

**Table 4:** Correlation of smoking with cell death (buccal epithelium) and genotoxicity (lymphocytes).

Cytogenetic damage	Basal	Chemotherapy	Radiotherapy
**Buccal epithelium**			
*Karyorrhexis*			
Yes	289.0 ± 79.6^b^	367.2 ± 63.0^b^	485.6 ± 81.0^abc^
No	239.1 ± 67.8	285.2 ± 72.1	451.3 ± 76.3^ac^
*Karyolysis*			*r* = 0.411^∗^ (*p* = 0.003)
Yes	86.2 ± 51.0	94.7 ± 61.3	293.3 ± 82.0^abc^
No	86.9 ± 54.8	105.3 ± 56.2	124.1 ± 69.6^a^
**Lymphocytes**			
*Damage index*			
Yes	152.8 ± 67.1	197.7 ± 58.6	208.4 ± 56.1^c^
No	145.7 ± 50.3	181.3 ± 57.4	214.9 ± 66.9^a^
*Frequency of damage*			
Yes	78.1 ± 20.1	88.7 ± 11.4	92.4 ± 8.5
No	81.2 ± 17.3	91.7 ± 8.8	88.8 ± 15.9


*Values represent the mean ± standard deviation. Significance up to *p* < 0.05. ^a^Compared to the chemotherapy group. ^b^Compared to individuals who answered “no.” ^c^Compared to baseline damage. ^∗^*p* values as compared to control group.*

**FIGURE 1:**
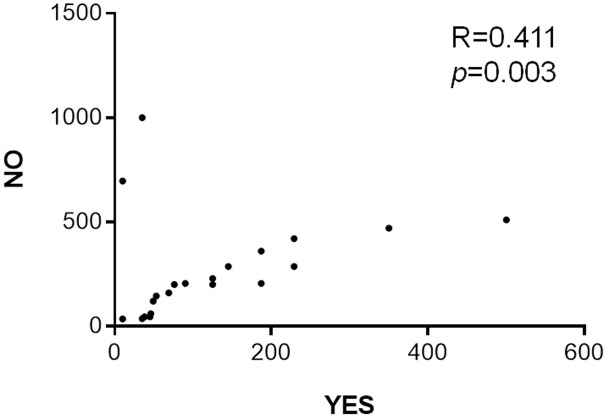
Correlation between smoking and karyolysis in buccal epithelium of breast cancer patients during radiotherapy. Spearman’s correlation ^∗^*p* < 0.05.

Smoking, a factor studied over the years with contradictory results, is currently recognized by the IARC as a carcinogenic agent with evidence of increased risk of breast cancer in humans (International Agency for Research on Cancer [IARC], 2015). Genotoxic evaluation using comet assay showed significant differences among smokers, non-smokers, and passive smokers, demonstrating induction of oxidative damages (Zalata et al., 2007). Positive correlations were observed between smoking and karyolysis in patients during RT (**Figure 1**). Moreover, studies also indicated the genotoxic effects of tobacco on oral epithelium (Tolbert et al., 1992; Trivedi et al., 1993) and mutagenic effects by micronuclei, karyorrhexis, karyolysis, and binucleate cells (Ergene et al., 2007). However, numerous reports indicate that there are no associations between smokers and increased DNA migration in peripheral lymphocytes of smokers of more than 20 cigarettes per day (Speit et al., 2003, 2007).

### Correlations between Smoking and Genetic Instability As Indicative of Cell Death and Genotoxicity in Breast Cancer Patients

Patients on QT and RT with alcohol consumption showed increased apoptosis levels in the buccal epithelium. Radiation therapy also increased the levels of biomarkers. Patients who reported consumption of alcoholic beverages at the baseline level did not show increase in DI and DF. However, during QT, these damages were significantly increased in patients who did not consume alcohol. No correlation was observed between ethylism and the cytogenetic biomarkers evaluated (**Table 5**). There is still limited evidence of smoking with the risk for breast cancer (Ghissassi et al., 2009; Secretan et al., 2009).

**Table 5:** Correlation of alcohol consumption with cell death (buccal epithelium) and genotoxicity (lymphocytes).

Cytogenetic damage	Basal	Chemotherapy	Radiotherapy
**Buccal epithelium**			
*Karyorrhexis*			
Yes	271.8 ± 93.5^b^	333.7 ± 83.1^b^	562.9 ± 79.3^abc^
No	212.1 ± 76.9	393.7 ± 82.7	439.5 ± 97.1^a^
*Karyolysis*			
Yes	109.7 ± 65.7^b^	123.1 ± 80.2^b^	219.2 ± 53.1^abc^
No	79.0 ± 46.7	91.6 ± 63.1	189.6 ± 87.8^a^
**Lymphocytes**			
*Damage index*			
Yes	147.5 ± 63.5	192.1 ± 59.8^b^	201.3 ± 45.1
No	149.5 ± 56.8	169.2 ± 32.1	215.1 ± 65.9^a^
*Frequency of damage*			
Yes	79.8 ± 19.2	89.3 ± 10.8	93.9 ± 4.7
No	77.7 ± 18.5	90.7 ± 9.9	89.5 ± 14.4


*Values represent the mean ± standard deviation. Significance up to *p* < 0.05. ^a^Compared to the chemotherapy group. ^b^Compared to individuals who answered “no.” ^c^Compared to baseline damage.*

Alcohol consumption is positively associated with breast cancer in menopausal women with more than 10 g/day (Lew et al., 2009). In postmenopausal women, there are associations between lifestyle (consumption of alcohol and tobacco) and inadequate diet with breast cancer. Thus, breast cancer preventive measures include no alcohol consumption (or only moderate), healthy diet, and physical activity (McKenzie et al., 2015). Interactions between lifestyle and dietary factors with genes are pointed out, for instance, the association with the MAPK genes such as MAPK 14 (p38) which may interact with alcohol, diet and lifestyle (Slattery et al., 2014). Although the biological mechanism has not yet been well elucidated, there is proven evidence that alcohol induces chromosomal instability resulting in aneuploidy events which are associated with cancer. In addition, the induction of oxidative damage, DNA adducts, crosslinks and DNA strand breaks can result in reactive oxygen species, lipid peroxidation product and acetaldehyde (Bonassi et al., 2011; Fenech and Bonassi, 2011). Interestingly the moderate consumption of alcohol reduced the risk of breast cancer by about 30% (Colditz and Bohlke, 2014; Howell et al., 2014).

### Correlations between Ionizing Radiation and Genetic Instability As Indicative of Cell Death and Genotoxicity

Patients were also reported to exposure of ionizing radiations (**Table 1**). At baseline, nuclear abnormalities of the buccal epithelium of patients who underwent RT and QT were significantly increased as compared to the control. RT also induced more cell death (karyorrhexis and karyolysis) when compared to QT. Positive correlations were observed between exposure to ionizing radiation and cell death by karyolysis in patients during QT as well as for DF at baseline and during QT as well (**Table 6**).

**Table 6:** Correlation of ionizing radiation with cell death (buccal epithelium) and genotoxicity (lymphocytes).

Cytogenetic damage	Control	Basal	Chemotherapy	Radiotherapy
**Buccal epithelium**				
*Karyorrhexis*				
Yes	172.3 ± 79.4^c^	250.9 ± 87.3^a^	355.2 ± 81.4^ad^	469.1 ± 169.7^abd^
No	154.8 ± 84.2	–	346.6 ± 73.9^a^	–
*Karyolysis*			*R* = 0.298^∗^ (*p* = 0.042)	
Yes	58.7 ± 45.0	86.7 ± 52.5^a^	107.5 ± 86.4^acd^	210.9 ± 83.2^abd^
No	45.3 ± 22.3	–	50.2 ± 15.5	–
**Lymphocytes**				
*Damage index*				
Yes	23.5 ± 18.1	157.0 ± 56.9^ac^	191.2 ± 56.9^ac^	211.8 ± 61.4^a^
No	29.1 ± 23.9	87.8 ± 25.3^a^	151.0 ± 29.7^a^	–
*Frequency of damage*		*R* = 0.279^∗^ (*p* = 0.050)	*R* = 0.391^∗∗^ (*p* = 0.005)	
Yes	14.7 ± 7.9	81.7 ± 18.7^ac^	91.6 ± 9.8^a^	90.6 ± 12.9^a^
No	16.3 ± 9.7	62.4 ± 5.7^a^	82.0 ± 7.7^a^	–


*Values represent the mean ± standard deviation. Significance up to *p* < 0.05. ^a^Compared to the control group. ^b^Compared to the chemotherapy group. ^c^Compared to individuals who answered “no.” ^d^Compared to baseline damage levels. ^∗^, ^∗∗^*p* values as compared to control group.*

Ionizing radiations are known to increase cancer risk (Preston et al., 2007; Kaiser et al., 2012) especially in patients undergoing further medical examinations (Kaiser et al., 2012). RT significantly increases DNA damage in lymphocyte in relation to QT and causes damage to repair genes (Synowiec et al., 2008). Exposure to X-rays induces significant increase in genotoxicity parameters (DI and DF) in peripheral blood lymphocytes of breast cancer patients compared to baseline levels in controls (**Table 6**). Ionizing radiation induces DNA damage and is known to be carcinogenic as it can cause DNA strand breaks as well as chromosomal rearrangements (Bekker-Jensen and Mailand, 2010). Double strand breaks are the most deleterious lesions induced by ionizing radiations which can lead to cell death due to their acute toxicities (Barnard et al., 2013). In addition to strand breaks, ionizing radiations induce damage to the chromosomal DNA, abasic sites, base and sugar oxidations, and cross-links (Eccles et al., 2011).

Exposure of DNA to radiation induces a signal transduction cascade resulting in damage to the genetic material, DNA strand breaks, including increased reactive oxygen species (Mantena et al., 2008; Mello et al., 2011; Taylor and Kirby, 2015), and induction of chromosomal aberrations and apoptosis (Szumiel and Foray, 2011). Tumor suppressor genes such as p53 and PTEN can be deregulated resulting in impairment of important functions such as induction of apoptosis, activation of the repair system and cell cycle arrest (Heinloth et al., 2003). RT increases the survival rate in cancer patients (Gentile et al., 2015) but may lead to clinical complications (Taylor and Kirby, 2015.) like genetic instability (Eidemüller et al., 2015). The effects and low doses of radiation are still uncertain but in women with germline mutations in *BRCA1* and *BRCA2* genes, an association with radiological diagnoses exists especially after 50 years of age (John et al., 2013).

## Conclusion

At the baseline, breast cancer patients already showed increased DNA damage in the oral epithelium (karyorrhexis and karyolysis) and lymphocytes (DI and DF) in relation to the control group. Our result indicating genetic instability were significantly increased during cancer therapies (QT and RT). The cytogenetic damages assessed during RT were bigger than those evidenced during QT. Cytogenetic damages were differentiated for risk factors (age, workplace, smoking, alcoholism, and exposure to X-rays) for genetic instability. Considering the age as risk factor for nuclear abnormalities indicative of cell death (karyorrhexis and karyolysis) were more evident in younger women and during the menopausal period as compared to women aged over 60 years. Negative correlations were found between age and nuclear abnormalities at the baseline level. These relationships were not observed for genotoxic damage in lymphocytes. Patients who reported smoking habit presented more karyorrhexis and karyolysis in the buccal epithelium; and genotoxicity in lymphocytes before (baseline) and during QT and RT. However, positive correlations were evidenced only between smoking and karyolysis for patients undergoing RT.

## Author Contributions

MP, MdA, AGJ, and KedCM performed the experimental work. MI, EA, MS, and MA performed the primary data analysis. SU, AdM, RdC, KádCM, AS, FdS, JdCS, DA, PF, SM, and AdCM-C performed the secondary data analysis and manuscript preparation. MI, JdS, AdCM-C, and SM wrote the manuscript. MI and SM communicated the research work.

## Conflict of Interest Statement

EA was employed by a company “Gaco Pharmaceuticals and Research Laboratory, Dhaka.”

The other authors declare that the research was conducted in the absence of any commercial or financial relationships that could be construed as a potential conflict of interest.
